# Double vitrification and warming does not compromise the chance of live birth—a potential invalid conclusion

**DOI:** 10.1093/hropen/hoad049

**Published:** 2024-01-04

**Authors:** Laszlo Nanassy, Beate Schoepper, Askan Schultze-Mosgau, Marion Depenbusch, Tanja K Eggersmann, Roman A F Hiller, Georg Griesinger

**Affiliations:** Universitäres Kinderwunschzentrum Luebeck, Luebeck, Germany; Universitäres Kinderwunschzentrum Luebeck, Luebeck, Germany; Department of Gynecological Endocrinology and Reproductive Medicine, University Hospital of Schleswig-Holstein, Luebeck, Germany; Universitäres Kinderwunschzentrum Luebeck, Luebeck, Germany; Department of Gynecological Endocrinology and Reproductive Medicine, University Hospital of Schleswig-Holstein, Luebeck, Germany; Universitäres Kinderwunschzentrum Luebeck, Luebeck, Germany; Department of Gynecological Endocrinology and Reproductive Medicine, University Hospital of Schleswig-Holstein, Luebeck, Germany; Universitäres Kinderwunschzentrum Luebeck, Luebeck, Germany; Department of Gynecological Endocrinology and Reproductive Medicine, University Hospital of Schleswig-Holstein, Luebeck, Germany; Universitäres Kinderwunschzentrum Luebeck, Luebeck, Germany; Department of Gynecological Endocrinology and Reproductive Medicine, University Hospital of Schleswig-Holstein, Luebeck, Germany; Universitäres Kinderwunschzentrum Luebeck, Luebeck, Germany; Department of Gynecological Endocrinology and Reproductive Medicine, University Hospital of Schleswig-Holstein, Luebeck, Germany

Sir,

We read with great interest the article by Makieva *et al.* (2023), an analysis of the association of double vitrification and warming with outcomes of embryo transfer. Given the worldwide increase of the cryopreservation of oocytes, zygotes and embryos in the context of routine ART, double vitrification is also increasing in frequency within some treatment paths (e.g. usage of vitrified autologous or donated oocytes for IVF or freeze-all cycles with zygotes and later surplus embryo freezing). Accordingly, the effects of double cryopreservation deserve special attention. Of note, only observational data are available to study the association of multiple cryopreservation events with treatment efficacy and safety. The foremost challenge of causal inference from observational data is controlling for confounding. In the study of Makieva *et al.* (2023), however, we suggest that potential key confounders have either been dismissed or not appropriately controlled for.

Briefly, frozen embryo replacement cycles were selected and the outcomes compared after single (blastocyst stage) or double (first at zygote, then blastocyst stage) vitrification and warming, respectively. The primary outcome was live birth rate per embryo transfer, which may constitute an inappropriate unit-of-analysis when individual patients with inherently different prognosis can contribute different numbers of embryo transfers to a pool of observations. Patients with a poorer prognosis may contribute more observations, thus negatively confounding the success rate per embryo transfer (Griesinger and Larsson, 2023). Indeed, the number of embryo transfers performed systematically differs between the interventions compared (310 FETs in 196 women (1.58) vs 97 FETs in 76 women (1.27) in the single vitrified-warmed (SVW) group vs the double vitrified-warmed (DVW) group, respectively). Moreover, a difference in prognosis has not sufficiently been explored, given the lack of data on treatment cycle rank, previous pregnancy, and, especially, pregnancy achievement in a fresh cycle before frozen embryo transfer (FET) cycle(s) (of note, 12.7% of women had FET more than two years after oocyte retrieval). Women with an indication for freeze-all are only present in the DVW group and these women may have a better prognosis, also evidenced by the higher number of oocytes collected.

It is not clear from the article what the reason is behind the difference in the number of embryo transfers per patient. It seems that the number of zygotes that are cultured to the blastocyst stage is equal in fresh and warmed cycles and randomly selected. Blastulation rates are also the same. One explanation is that culturing of warmed zygotes of better prognosis patients results in a higher chance of pregnancy and recryopreserved blastocysts are transferred at a lower frequency. Another possibility is that the culturing of warmed zygotes might result in fewer available blastocysts for cryopreservation either owing to lower quality blastocysts or an unintentional more thorough embryo selection process. It is also possible that double vitrified blastocysts are left as a last option for patients and there is a preference to thaw zygotes and culture these to the blastocyst stage, transferring a single cryopreserved (at the PN stage) blastocyst. It is, however, only a speculation without a detailed description of the transfer policy. Nevertheless, data analysis should consider and try to control such possible confounding.

We also want to point out that recent reports have put the assumption that Day 5 and Day 6 embryos have the same developmental potential under scrutiny (Ferreux *et al.*, 2018; Bourdon *et al.*, 2019; Yerushalmi *et al.*, 2021). This important confounder has also not been accounted for in the analysis.

Another issue is the use of statistical tests, such as chi-square, that require independent observations for a valid result. As outlined above, a pool of embryo transfers, where one individual can contribute different numbers of observations, does not constitute such independent observations and most *P* values presented must therefore be dismissed.

If the unit-of-analysis was the patient, the cumulative achievement of live birth from the FETs performed in the two groups would favor the single vitrification group (45.41% (89/196) vs 39.47% (30/76) in SVW and DVW groups, respectively). However, this picture is incomplete without knowledge of cycle rank and pregnancy achievement in the fresh transfer.

In summary, we ask Makieva *et al.* to please re-do their analysis on independent events (i.e. the first frozen embryo transfer cycle per women in two groups of women carefully controlled for equal prognosis, including pregnancy achievement in the fresh cycle) and/or using multi-variable analysis that controls for confounders and dependent events. This is necessary so as to corroborate or refute the conclusion of no-harm of double vitrification and avoiding overutilization under a wrong assumption.

## References

[hoad049-B1] Bourdon M , Pocate-Cheriet K, Finet de Bantel A, Grzegorczyk-Martin V, Amar Hoffet A, Arbo E, Poulain M, Santulli P. Day 5 versus day 6 blastocyst transfers: a systematic review and meta-analysis of clinical outcomes. Hum Reprod 2019;34:1948–1964. 31644803 10.1093/humrep/dez163 PMC7967799

[hoad049-B2] Ferreux L , Bourdon M, Sallem A, Santulli P, Barraud-Lange V, Le Foll N, Maignien C, Chapron C, de Ziegler D, Wolf JP et al Live birth rate following frozen-thawed blastocyst transfer is higher with blastocysts expanded on Day 5 than on Day 6. Hum Reprod 2018;33:390–398. 29394365 10.1093/humrep/dey004

[hoad049-B3] Griesinger G , Larsson P. Conventional outcome reporting per IVF cycle/embryo transfer may systematically underestimate chances of success for women undergoing ART: relevant biases in registries, epidemiological studies, and guidelines. Hum Reprod Open 2023;2023:hoad018. 37250429 10.1093/hropen/hoad018 PMC10214861

[hoad049-B4] Makieva S , Sachs MK, Xie M, Velasco A, El-Hadad S, Kalaitzopoulos DR, Dedes I, Stiller R, Leeners B. Double vitrification and warming does not compromise the chance of live birth after single unbiopsied blastocyst transfer. Hum Reprod Open 2023;2023:hoad037. 37840636 10.1093/hropen/hoad037 PMC10576635

[hoad049-B5] Yerushalmi GM , Shavit T, Avraham S, Youngster M, Kedem A, Gat I, Dorofeyeva US, Mashiach S, Schiff E, Shulman A et al Day 5 vitrified blastocyst transfer versus day 6 vitrified blastocyst transfer in oocyte donation program. Sci Rep 2021;May 2111:10715. 34021226 10.1038/s41598-021-90238-y PMC8139971

